# Research on the mechanism of the influence of family socioeconomic status on the Cardinal principle of young children

**DOI:** 10.1038/s41598-025-04693-y

**Published:** 2025-06-05

**Authors:** Huanhuan Li, Bingyu Duan, Mengzhen Luo, Mingci Chen, Enwei Xu

**Affiliations:** 1 College of Educational Science, Xinjiang Normal University, Urumqi, 830017 China; 2 School of Education Science, Hanshan Normal University, Chaozhou, 521041 China

**Keywords:** Family socioeconomic status, Cardinality principle, Executive function, Approximate number system, Receptive vocabulary skills, Psychology, Human behaviour

## Abstract

This study investigated the mediating roles of Executive Function, Approximate Number System, and Receptive Vocabulary Skills in Family Socioeconomic Status and Cardinality Principle. A cross-sectional research design was used in this study. The study included 130 young children (63 boys and 67 girls, mean age = 68.52 ± 7.37 months) and their parents. Correlation analyses revealed significant positive correlations between children’s understanding of the Cardinality Principle and Family Socioeconomic Status, Executive Function, Approximate Number System, and Receptive Vocabulary Skills. After controlling for children’s gender and age, mediation analyses indicated that Family Socioeconomic Status significantly and positively affected children’s base comprehension. At the same time, Executive Function, Receptive Vocabulary Number System, and Receptive Vocabulary Skills all mediate the relationship between Family Socioeconomic Status and children’s Cardinality Principle to some extent.

The cardinal principle (CP) states that individuals can understand that the last number reflects the total number of items in a set when counting^1^. Once children have mastered CP, they can meaningfully interpret any number mentioned during counting^2^. CP is a key milestone in the development of children’s number concepts and an important core indicator for predicting subsequent mathematical achievements^3,4^. Studies have shown that early CP comprehension can significantly predict first-grade math scores^1^performance in number system knowledge^5^complexity of arithmetic strategies used, and problem-solving skills^6^. Therefore, revealing the influence mechanism of CP has important practical value for early mathematical intervention.

Recent research has increasingly highlighted the associations among family socioeconomic status (SES), young children’s executive function (EF), approximate number system (ANS), and receptive vocabulary skills (RVS) with the development of CP. However, findings in the existing literature regarding the relationships among these factors and CP are inconsistent. Some studies indicate that SES significantly impacts young children’s CP^7,8^while others suggest that this effect may be mediated by children’s cognitive abilities^9,10^. Similarly, diverse studies offer varying evidence and interpretations regarding the roles of EF, ANS, and RVS in CP development^11–15^.

Given these discrepancies, there is a pressing need for a more comprehensive and systematic exploration of the relationships between SES and young children’s CP development, particularly focusing on the mediating roles of EF, ANS, and RVS. While previous studies have provided valuable insights for future research, several shortcomings remain. First, the applicability of the relationships between SES and CP within the Chinese cultural context has yet to be established. Second, existing literature lacks a standardized examination of the relationships among EF, ANS, RVS, and CP. Moreover, few studies have examined the parallel multiple mediations of EF, ANS, and RVS when SES influences CP in young children. To address these gaps, this study collected data on young children’s SES, EF, ANS, RVS, and CP, analyzing it using SPSS 29.0 and the PROCESS plug-in to explore the direct and indirect relationships of SES on CP. Specifically, this study aims to answer the following questions:

- How is SES related to the development of CP in young children in China?
- How are EF, ANS, and RVS related to young children’s CP?
- Can parallel multiple mediation models with EF, ANS, and RVS acting as independent mediators explain the relationship between SES and CP acquisition in young children?

## Effects of family socioeconomic status on young children’s cardinality principle

SES is a multidimensional construct reflecting a family’s access to and control of various social resources. It is often expressed through the social capital of family members, encompassing aspects such as power and prestige, thereby highlighting individual differences in access to actual and potential resources^16^. Various assessment criteria have been employed in academic research to determine young children’s SES. Despite the diversity of these approaches, it is common practice to evaluate SES based on indicators such as family income, parental education levels, and occupational status^17^.

The impact of SES on young children’s early mathematical learning is significant. Numerous studies have demonstrated that children from lower SES backgrounds face various challenges in learning mathematics, including difficulties in generating interest, building confidence, acquiring mathematical knowledge, and applying advanced problem-solving skills^18–20^. These challenges undermine their mathematical abilities and exacerbate existing inequalities in educational achievement^21^. The roles of SES are particularly evident in early childhood, especially regarding learning the cardinality principle (CP). Research has consistently shown that preschoolers from low-income families lag behind their higher SES peers in CP understanding even before formal schooling begins, mainly due to their limited exposure to math-related resources and activities at home^7,17,22,23^. This early learning gap may further amplify deficits in mathematical skills as children enter formal education^8^. Consequently, we propose the following research hypothesis:

**Hypothesis 1 (H1):** Young children’s CP level increases with SES.

## Executive function as a mediator of family socioeconomic status and young children’s cardinality principle

EF encompasses a set of cognitive abilities that facilitate goal-directed behaviours essential for effective academic and daily life performance^24^. This construct includes components such as inhibitory control, working memory, and cognitive flexibility, all of which emerge in early childhood and continue to develop into adulthood^25,26^. These skills are crucial for self-regulation, attention, and task-switching, all of which play foundational roles in the learning process, particularly in mathematics^27,28^. The association between EF and mathematical competence is well-established^29–31^. However, research linking EF to CP in young children remains relatively sparse. Recent neuroscientific studies have identified critical brain regions, including the prefrontal, orbitofrontal, and anterior cingulate cortices, as vital for maintaining EF^32^. Additionally, the occipital-parietal-frontal network plays a significant role in number processing^33^suggesting shared physiological and cognitive mechanisms between EF and CP. While some studies indicate a positive predictive relationship between EF and young children’s mastery of CP^11,34^others report no significant connection^12,35^.

The role of EF in bridging the gap between SES and academic achievement has garnered increasing attention^21,36,37^particularly in the context of developing mathematical skills^38–40^. For instance, a study found that performance on the Tower of Hanoi task (an EF indicator) mediated the relationship between income and math and reading achievements for third graders^38^. Another study demonstrated that EF mediated the association between SES and math achievement even after controlling for verbal abilities^41^. Further research by Lawson and Farah^39^ confirmed the partial mediating role of EF between SES and changes in math achievement. Moreover, Waters et al.^40^ found that working memory, as a component of EF, mediated the relationship between parental education and young children’s math achievement. Based on these findings, we hypothesize that EF is closely linked to CP and may mediate the relationship between SES and CP learning, although this hypothesis requires further empirical investigation.

**Hypothesis 2 (H2):** EF partially mediates the relationship between SES and CP.

## Approximate number system as a mediator of family socioeconomic status and young children’s cardinality principle

The ANS has a complex relationship with the concept of cardinality. ANS enables individuals to make rapid, approximate quantitative judgments without relying on precise counting or symbolic numbers^42^. Numerous studies highlight the importance of ANS for understanding CP. Cognitive neuroscience research has identified the intraparietal sulcus—a key brain region associated with ANS—as critical for numerical cognition, suggesting a link between ANS and CP^43^. Empirical evidence further supports that ANS is essential for developing CP in young children^12,13,44,45^. However, some studies present opposing views; for example, Schröder et al.^46^ found that ANS acuity at 1.5 years did not significantly influence the later acquisition of CP. Additionally, some researchers argue that ANS and mathematical skills may function independently^47–49^.

Despite these conflicting perspectives, most evidence suggests that ANS facilitates young children’s understanding of early mathematical concepts^50–52^. Young children with higher ANS acuity are more likely to grasp symbolic quantitative knowledge, such as cardinal number understanding and computational skills^53–55^. Traditionally, the development of ANS has been thought to be primarily influenced by genetic and biological factors^56^. However, recent research indicates that environmental factors, particularly SES, can significantly impact young children’s ANS performance^10,57,58^. For example, McNeil et al.^57^ found that children from moderate to high SES backgrounds exhibited significantly higher ANS acuity than their low SES counterparts. Similarly, Bachman et al.^10^ confirmed a positive correlation between SES and ANS accuracy.

Conversely, some studies have found no significant associations between parental education, household income, and ANS accuracy in preschool samples^59–61^. These discrepancies may stem from the reliance on single SES indicators^10^. The current research on the relationship between SES and ANS remains inconsistent, and existing studies often lack a comprehensive approach to SES measurement. Moreover, the interplay between SES and ANS has been underexplored in the context of Chinese culture. Therefore, this study aims to reexamine the relationships among SES, ANS, and CP in Chinese preschoolers, employing a more comprehensive set of SES indicators, including family income, parental educational background, and occupational information. Considering the potential link between SES and ANS, along with the role of ANS in young children’s learning of mathematical concepts, we propose the following hypothesis:

**Hypothesis 3 (H3): **ANS partially mediates the relationship between SES and CP.

## Receptive vocabulary skills as a mediator of family socioeconomic status and young children’s cardinality principle

RVS refer to an individual’s ability to understand and recognize vocabulary^62^ and has gained increasing attention in the context of young children’s mathematics learning. Language skills are considered indispensable for mathematics acquisition^63^prompting researchers to explore the potential link between RVS and the cardinality principle (CP).

Several theoretical frameworks emphasize the crucial role of RVS in young children’s comprehension of mathematical terms and concepts. For instance, Barner^64^ argues that young children learn to utilize number concepts by first mastering singular and plural forms, such as “one dog” and “two dogs.” Similarly, Spelke^65^ posits that the development of noun phrases (e.g., “two rabbits”) is essential for understanding the meaning of the initial number words. Other scholars suggest that stronger RVS enhances young children’s ability to access math-related linguistic information during social interactions, which subsequently enriches their mathematical terminology and deepens their conceptual understanding^66,67^. However, empirical findings on this relationship have been inconsistent^14,15,68^. For example, Pixner et al.^68^ found a significant correlation between young children’s understanding of base ten numbers and their vocabulary skills. Chow and Ekholm^15^ reported that RVS did not predict young children’s math achievement.

RVS may serve as a mediating factor between SES and young children’s CP development. Research indicates that children from high-SES families generally outperform their peers from low to moderate-SES backgrounds in vocabulary skills^69–72^. The Family Investment Model suggests that high-SES parents are better positioned to provide their children with rich educational resources and high-quality learning environments^73–75^. Furthermore, SES disparities are evident in the quality of parent-child interactions—such as communication, sensitivity, and supportiveness—and the availability of learning resources, including books and toys^76,77^. These factors significantly influence young children’s vocabulary development.

The interplay between SES, vocabulary, and mathematical proficiency is critical. Poor language development can hinder foundational early math knowledge acquisition, particularly for children from low-SES backgrounds^78^. Studies have shown that preschoolers from middle-SES families outperform their low-income peers on verbal forms of number combination and math story problems^79–81^. This advantage is attributed to the mastery of number vocabulary, which facilitates performance on mathematical tasks^82^. Deficits in number vocabulary among low-SES preschoolers may contribute to delays in their math skill development^83^.

Although SES, vocabulary, and young children’s math skills are strongly correlated, the specific relationships among SES, RVS, and young children’s CP have not been thoroughly investigated. Given the complex interactions among these variables, we hypothesize that RVS may elucidate the relationship between SES and the development of young children’s CP.

**Hypothesis 4 (H4): **RVS partially mediates the relationship between SES and CP.

**Fig. 1:**
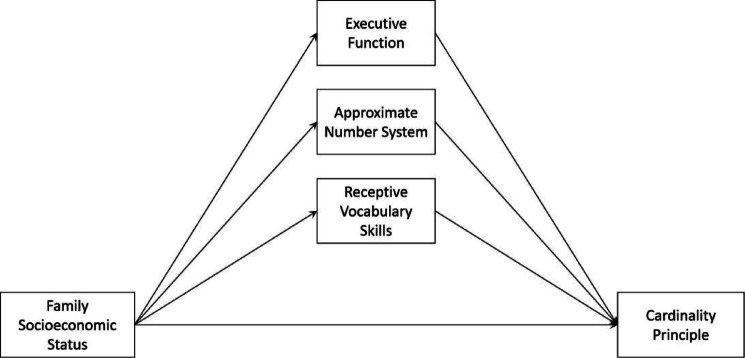
Theoretical model of mediation of EF, ANS, and RVS between SES and CP.

According to the research hypothesis proposed above, a multiple-parallel mediation model was constructed in this study, as shown in Fig. 1.

## Method

### Participants

The research team collaborated with several public kindergartens in Urumqi and Changji regions to conduct the study. Invitation letters were sent to parents throughout kindergarten to introduce the study’s purpose, process, and benefits. At the same time, recruitment information and contact information were posted in the online community of kindergarten parents. The teacher calls the invitation for parents who cannot access online information. Participants had to have children between the ages of 3 and 7 years; No intellectual or hearing impairment; Vision was normal or corrected. Families were initially screened at the time of recruitment and further invited if they met the criteria. A total of 145 children were recruited. Their parents completed SES information through online and offline questionnaires, and 145 valid questionnaires were collected. In addition to the three key items used to assess SES: parental education level, occupation and annual household income, the questionnaire also contains some questions to collect basic demographic information, such as children’s gender and age. After the exclusion of absence due to illness, scheduling conflicts, and incomplete data, 130 valid cases (63 males, 67 females, mean age 68.52 months, SD = 7.367) were finally used for analysis. SES was assessed according to parental education, family income, and occupation, as shown in Table 1.

**Table 1:** The sample characteristics.

Variable	Categories	*N*	Percentage (%)
Parents’ education level	Elementary or below	1	0.8
	Junior high school	5	3.8
	Junior college, vocational high school, or high school technical school	13	10.0
	College	32	24.6
	Undergraduate	68	52.3
	Graduate students and above	11	8.5
Average gross monthly household income	Less than 2,000 Yuan	8	6.2
	2001–5000 Yuan	35	26.9
	5001–8000 Yuan	37	28.5
	8001–10,000 Yuan	22	16.9
	Above 10,000 Yuan	28	21.5
Parents’ occupation	Temporary workers, unemployed, unemployable, unskilled, and agricultural labourers	9	6.9
	Career senior executives (managers) with professional technicians, professional supervisors (party leaders)	30	23.1
	Middle-level managers with middle-level professional and technical staff, assistant professional staff	64	49.2
	Manual labour workers (commercial service workers), self-employed, skilled workers, and workers of the same level	17	13.1
	General managers with general professional and technical staff, transactional staff	10	7.7
Total		130	100

### Measures

#### Family socioeconomic status (SES)

Drawing on Ren’s^84^ methodology, this study assesses SES using parental education level, occupation, and annual household income as indicators. First, quantitative values are assigned to the three indicators, which are then converted into a standardized score, *Z*. Next, principal component analysis is used to determine the factor loadings of the indicators. The formula is SES = (*β*1*Z Parental Education level + *β*2*Z Parents’ occupation + *β*3*Z Average gross monthly household income)/*εf*. *β* represents the factor loadings of each indicator, and *εf* is the eigenvalue of the first factor. A high SES score indicates a high SES in the family. In the present study, Cronbach’s *α* of the scale was 0.700.

#### Executive function

The assessment of EF is based on the average score of the three tasks. Specifically, scores were obtained by the day-night Stroop task, digital recall task, and Dimension variation card sorting test (DCCS), and then the average scores for these three tasks were calculated as the total EF score.

Inhibitory Control: The Day-Night Stroop Task was utilised as devised by Marcovitch et al.^85^. At the beginning of the experiment, participants were shown pictures of the sun and the moon and helped to establish the concepts of “sun for day” and “moon for night”. The participants were then told the task rules: “night” when they saw the sun and “day” when they saw the moon. The experimental material consisted of 10 pictures of the sun and 10 pictures of the moon, and the two pictures were presented randomly. Participants were given five practice sessions to familiarize themselves with the rules of the task, and 20 formal tests were performed after the sessions. One point is awarded for each correct answer, and no point is awarded for each incorrect answer. The total score ranges from 0 to 20 points.

Working Memory: The Digit Recall task was adapted from Gathercole et al.^86^. Before the test, the task rules were explained to the subjects: the tester read the numbers at one number per second, asked the subjects to recall the numbers in order, and led the subjects to practice 1–2 lists. The formal test starts with a List of Lengths 1, and if the subject correctly recalls four groups of numbers of the same length, the test proceeds to the following List of Lengths. The test is stopped if the subject continuously incorrectly recalls three sets of numbers of the same length. The test consists of 7 lists ranging in length from 1 to 7 digits, and each List length contains 3 groups of numbers, each group of numbers being non-repetitive random numbers from 1 to 9. One point is awarded for correct answers, no points are awarded for incorrect answers, and the total score ranges from 0 to 21 points.

Cognitive Flexibility: This subtask is based on the Dimension Change Card Sorting Task (DCCS) designed by Zelazo^87^. Before the test, the participants were shown pictures of red and blue cats and boats and told that the cards with the same colour should be grouped when classifying by colour. When sorting by shape, cards with the same shape must be grouped. Before the formal test, the subjects were led to conduct 6 practice tests: classified by “shape” 3 times and classified by “colour” 3 times. The formal test consisted of 2 groups, each required to be classified by “shape” and “colour” 12 times, 24 times. One point is awarded for correct answers, no points are awarded for incorrect answers, and the total score ranges from 0 to 24 points.

#### Approximate number system

ANS was measured using Halberda et al.’s^44^ Panamath software, where children compared quantities of yellow and blue dots. They had 3000 milliseconds to determine which colour had more dots. The task consisted of 8 practice tests and 80 formal tests, of which 40 tests had more yellow dots and 40 tests had more blue dots. After the test is over, the software will automatically generate test results, namely response time, accuracy rate and Weber coefficient, among which accuracy is the main criterion^45,88^. This test method has been proven suitable for Chinese children^88^.

#### Receptive vocabulary skills

RVS were assessed using the Peabody Picture Vocabulary Test-Revised (PPVT-R), modified by Guo et al.^89^. Participants were trained three times before the formal assessment, which involved selecting pictures corresponding to words read in Mandarin. Each correct answer received one point, while incorrect responses received no points. The test comprised 120 questions and was discontinued if the child made six consecutive errors. The final score was calculated as the number of correct answers minus the number of incorrect answers.

#### Cardinality Principle

By combing the existing studies, it is found that the paradigms for assessing children’s CP roughly include verbal counting, number trial, How Many task and Give-N task. However, compared with verbal counting and number recognition, the How Many and Give-N tasks are more common, more accurate, and more effective ways to test children’s use of CP^90^. For example, Paliwal and Baroody^91^ used the How Many and Give-N task to assess CP development in young children aged 2–4 years. Therefore, in this study, children were tested with the How many and Give-N task to assess their CP development. The study employed the How Many and Give-N tasks to assess CP, based on Zhao^92^ and Zhang^93^. CP scores are calculated based on the average of How Many and Give-N task scores. In the How Many Task, the tester presented blocks and asked children to count them and state the total. In the Give-N Task, the tester instructed the children to retrieve a specified number of blocks. Both tasks were administered 9 times, with each correct response scored as 1 point and incorrect responses not scored. The scoring range for each task was 0–9 points. In this study, Cronbach’s α was 0.850 for How Many tasks and 0.885 for Give-N tasks. These values indicate acceptable internal consistency and reliability for both tasks.

#### Covariates

Numerous studies have shown that young children’s age is closely related to the development of EF^94^ANS^44^vocabulary acquisition^95^and CP^96^. Secondly, the gender difference in the early cognitive domain has been confirmed by many studies. Some research suggests that boys may temporarily be advantageous in number-related tasks^97^while girls tend to outperform in EF tasks^98^. Given the complex associations of age and sex with EF, ANS, RVS, and CP, this study aimed to control for possible confounding by young children’s age and gender and, thus, more precisely explore the relationship between SES and CP. To ensure the accuracy and reliability of the research results and to reveal the internal relationship between various factors in more depth.

#### Ethical approval and consent to participate

The study was conducted following the Declaration of Helsinki. The authors obtained informed consent from all children’s parents or legal guardians to participate in the research, and informed consent was obtained from a parent and/or legal guardian. Each participant could withdraw from the study at any stage of the project. The research was conducted following relevant guidelines and regulations with the consent and under the University Committee on Human Research Protection supervision at Shanghai Normal University No. 2,023,035.

Data collection occurred in May 2024 and was conducted by six professionally trained researchers. Each child was tested individually in a quiet environment for approximately 25 to 40 min, with all instructions delivered in Mandarin. After the assessment, participants received stickers as a reward.

### Data analysis

This study used SPSS 29.0 software and SPSS PROCESS v4.1 plug-in to analyze the data statistically. First, descriptive statistics and correlation analysis were used to explore the interrelationships among the variables. Then, mediation role tests were conducted based on the traditional stepwise regression method, with statistical significance at *p* < 0.05, *p* < 0.01 and *p* < 0.001. In addition, a nonparametric bootstrap method (5000 samples) was utilized to assess the significance of the total and indirect relationships. This study examines the mechanism of the influence of SES on CP using EF, ANS, and RVS as mediating variables and constructs a parallel multiple mediation model.

1 $$\:\:\text{C}\text{P}={\alpha\:}_{0}+c\text{S}\text{E}\text{S}+{\alpha\:}_{1}\text{c}\text{o}\text{n}\text{t}\text{r}\text{o}\text{l}+{\epsilon\:}_{1}$$

2 $$\:\text{E}\text{F}={\beta\:}_{0}+{\beta\:}_{1}\text{S}\text{E}\text{S}+{\beta\:}_{2}\text{c}\text{o}\text{n}\text{t}\text{r}\text{o}\text{l}+{\epsilon\:}_{2}$$

3 $$\:\text{A}\text{N}\text{S}={\gamma\:}_{0}+{\gamma\:}_{1}\text{S}\text{E}\text{S}+{\gamma\:}_{2}\text{c}\text{o}\text{n}\text{t}\text{r}\text{o}\text{l}+{\epsilon\:}_{3}$$

4 $$\:\text{R}\text{V}\text{S}={\delta\:}_{0}+{\delta\:}_{1}\text{S}\text{E}\text{S}+{\delta\:}_{2}\text{c}\text{o}\text{n}\text{t}\text{r}\text{o}\text{l}+{\epsilon\:}_{4}$$

5 $$\:\text{C}\text{P}={\eta\:}_{0}+c{\prime\:}\text{S}\text{E}\text{S}+{\eta\:}_{1}\text{E}\text{F}+{\eta\:}_{2}\text{A}\text{N}\text{S}+{\eta\:}_{3}\text{R}\text{V}\text{S}+{\eta\:}_{4}\text{c}\text{o}\text{n}\text{t}\text{r}\text{o}\text{l}+{\epsilon\:}_{5}$$.

In the model, CP stands for the cardinality principle, SES stands for family socioeconomic status, EF, ANS, and RVS stands for executive function, approximate number system, and receptive vocabulary skills, respectively, and control denotes the control variables. *α*_*0*_, *β*_*0*_, *γ*_*0*_, *δ*_*0*_ and *η*_*0*_ are the intercept terms. *α*_*1*_, *β*_*2*_, *γ*_*2*_, *δ*_*2*_ and *η*_*4*_ are the impact coefficients. *ε*_*1*_, *ε*_*2*_, *ε*_*3*_, *ε*_*4*_ and *ε*_*5*_ are the residuals. Model (1) analyzes the direct effect of SES on the CP, where *c* is the total impact coefficient. Model (2) examines the effect of SES on EF, where *β*_*1*_ is the influence coefficient. Model (3) examined the impact of SES on the ANS, with *γ*_*1*_ as the impact coefficient. Model (4) examined the effect of SES on RVS, with *δ*_*1*_ as the influence coefficient. Model (5) examined the effect of SES on CP, controlling for EF, ANS, and RVS, with *c*′ as the direct influence coefficient. *η*_*1*_, *η*_*2*_ and *η*_*3*_ are the influence coefficients of EF, ANS, and RVS on CP, respectively. The independent mediation effect consists of the following three paths: “SES → EF → CP”(Path 1), “SES → ANS → CP”(Path 2), and “SES → RVS → CP”(Path 3). The mediating effect values were *β*_*1*_*η*_*1*_, *γ*_*1*_*η*_*2*_, and *δ*_*1*_*η*_*3*_, and the sum of the three was the total mediating effect. Therefore, the total effect c of SES on CP consists of the direct effect c′ and the mediating effect together, i.e., *c* = *c*′ + *β*_*1*_*η*_*1*_ + *γ*_*1*_*η*_*2*_ + *δ*_*1*_*η*_*3*_. In addition, a partial mediation effect was observed if the total effect c was significant and at least one of the mediating paths (e.g., EF, ANS, or RVS) was also significant. If the total effect *c* was significant, all mediating paths were significant, and the direct effect c’ was not significant, it was a fully mediated effect.

## Results

### Descriptive statistics and correlation among the studied variables

Table 2 shows the results of descriptive statistics and correlation analyses for each variable. Age was associated with EF (*r* = 0.306, *p* < 0.001), ANS (*r* = 0.225, *p* < 0.05), RVS (*r* = 0.401, *p* < 0.001) and CP (*r* = 0.544, *p* < 0.001). In terms of gender, there was no significant correlation between each gender variable. The CP was positively correlated with SES (*r* = 0.432, *p* < 0.001), inhibitory control (*r* = 0.360, *p* < 0.001), working memory (*r* = 0.454, *p* < 0.001), cognitive flexibility (*r* = 0.423, *p* < 0. 001), total EF scores (*r* = 0.570, *p* < 0.001), ANS (*r* = 0.503, *p* < 0.001) and RVS (*r* = 0.571, *p* < 0.01) were positively correlated. Also, scores on two subtasks of the CP, how many and give-n, were significantly and positively correlated with these variables. Second, positive correlations were also found between SES and inhibitory control (*r* = 0.329, *p* < 0.001), working memory (*r* = 0.236, *p* < 0.01), cognitive flexibility (*r* = 0.316, *p* < 0.001), total EF scores (*r* = 0.413, *p* < 0.001), ANS (*r* = 0.261, *p* < 0.01), and RVS (*r* = 0.303, *p* < 0.001). This provides the basis for mediation effect tests. In addition, the sample was divided into high and low groups by SES level using the median as the cut-off point. Subsequently, an independent samples t-test was used to analyze the differences in EF, ANS, RVS and CP between the two groups, aiming to test whether the above variables were statistically significant at different SES levels. According to the data in Table 3, there were statistically significant differences between different SES levels of young children in EF (*t* =−4.241, *Cohen’s d*= −0.748, *p* < 0.001), ANS (*t* =−2.930, *Cohen’s d*= −0.963, *p* < 0.01), RVS (*t* =−2.875, *Cohen’s d*= −0.504, *p* < 0.01), and CP (*t* = −5.153, *Cohen’s d*= −0.905, *p* < 0.001) were all statistically different from each other, suggesting that the EF, ANS, RVS, and CP scores of the high SES group were significantly higher than those of the low SES group.

**Table 2:** Means, standard deviations, and correlations among variables.

	Mean	SD	1	2	3	4	5	6	7	8	9	10	11
1. SES	−0.001	3.000	1										
2. Age	68.520	7.367	0.274**	1									
3. IC	18.550	3.353	0.329***	0.200*	1								
4. WM	14.020	2.857	0.236**	0.248**	0.198*	1							
5. CF	22.280	3.473	0.316***	0.216*	0.358***	0.248**	1						
6. EF	18.285	2.321	0.413***	0.306***	0.741***	0.629***	0.773***	1					
7. ANS	86.562	10.877	0.261**	0.225*	0.547***	0.261**	0.381***	0.561***	1				
8. RVS	55.250	19.019	0.303***	0.401***	0.317***	0.387***	0.361***	0.491***	0.350***	1			
9. HM	6.860	2.452	0.434***	0.553***	0.301**	0.384***	0.417***	0.510***	0.431***	0.551***	1		
10. GN	6.290	2.799	0.380***	0.490***	0.369***	0.461***	0.379***	0.556***	0.507***	0.522***	0.757***	1	
11. CP	6.577	2.466	0.432***	0.544***	0.360***	0.454***	0.423***	0.570***	0.503***	0.571***	0.928***	0.946***	1

*Notes.* ^*^p < 0.05, ^**^p < 0.01, ^***^p < 0.001. Only significant data are shown in the table. SES: family socioeconomic status; IC: inhibitory control; WM: working memory; CF: cognitive flexibility; EF: executive function; ANS: approximate number system; RVS: receptive vocabulary skills; HM: How many; GN: Give-n; CP: cardinality principle. The same applies to the tables in this paper.

**Table 3:** Independent sample t-test for the effect of different levels of SES on EF, ANS, RVS, and CP.

Variable	Low SES group (*n* = 64)		High SES group (*n* = 66)		Independent sample t-test		
	Mean	SD	Mean	SD	t	*p*	Cohen’s d
IC	17.700	4.500	19.380	1.120	−2.892	0.005	−0.512
WM	13.410	2.910	14.620	2.690	−2.472	0.015	−0.432
CF	21.250	4.400	23.270	1.770	−3.419	0.001	−0.602
EF	17.453	2.814	19.091	1.296	−4.241	0.000	−0.748
ANS	83.784	12.972	89.260	7.530	−2.930	0.004	−0.963
RVS	50.520	19.450	59.850	17.550	−2.875	0.005	−0.504
HM	5.800	2.720	7.890	1.600	−5.337	0.000	−0.937
GN	5.280	2.970	7.270	2.240	−4.306	0.000	−0.757
CP	5.540	2.690	7.580	1.710	−5.153	0.000	−0.905

### Test for multiple parallel mediation

In this study, multivariate stratified regression analyses were conducted using the SPSS macro program Process developed by Hayes^99^which was used to test for mediating roles. According to the results of the correlation analysis, the age of children was included as a covariate in the mediation model analysis. The regression showed that SES significantly and positively predicted CP (*β* = 0.303, *t* = 4.211, *p* < 0.001), and the results were significant even after inputting EF, ANS, and RVS (*β* = 0.144, *t* = 2.219, *p* < 0.05). In addition, data from Models 1, 2, and 3 indicated that SES positively predicted EF (*β* = 0.355, *t* = 4.339, *p* < 0.001), ANS (*β* = 0.216, *t* = 2.461, *p* < 0.05), and RVS (*β* = 0.209, *t* = 2.536, *p* < 0.05). Thus, EF, ANS, and RVS partially mediate the relationship between SES and young children’s CP, respectively, as shown in Table 4.

**Table 4:** Regression analysis of the mediation model.

Significance of Regression Coefficients		Model 1 (EF)				Model 2 (ANS)				Model 3 (RVS)				Model 4 (CP)			
		*B*	*Se*	*t*	*β*	*B*	*Se*	*t*	*β*	*B*	*Se*	*t*	*β*	*B*	*Se*	*t*	*β*
Predictor Variables	SES	0.275	0.063	4.339***	0.355	0.784	0.318	2.461*	0.216	1.325	0.523	2.536*	0.209	0.118	0.053	2.219*	0.144
	EF	-	-	-	-	-	-	-	-	-	-	-	-	0.190	0.084	2.266*	0.179
	ANS	-	-	-	-	-	-	-	-	-	-	-	-	0.048	0.016	2.984**	0.211
	RVS	-	-	-	-	-	-	-	-	-	-	-	-	0.031	0.009	3.387**	0.239
	month	0.208	0.026	2.539*	0.208	0.244	0.130	1.881	0.165	0.888	0.213	4.169***	0.344	0.106	0.022	4.890***	0.316
Overall Fit Index	R	0.459				0.306				0.449				0.763			
	R^2^	0.211				0.094				0.201				0.582			
	F	16.926***				6.559**				16.004***				34.475***			

**Fig. 2:**
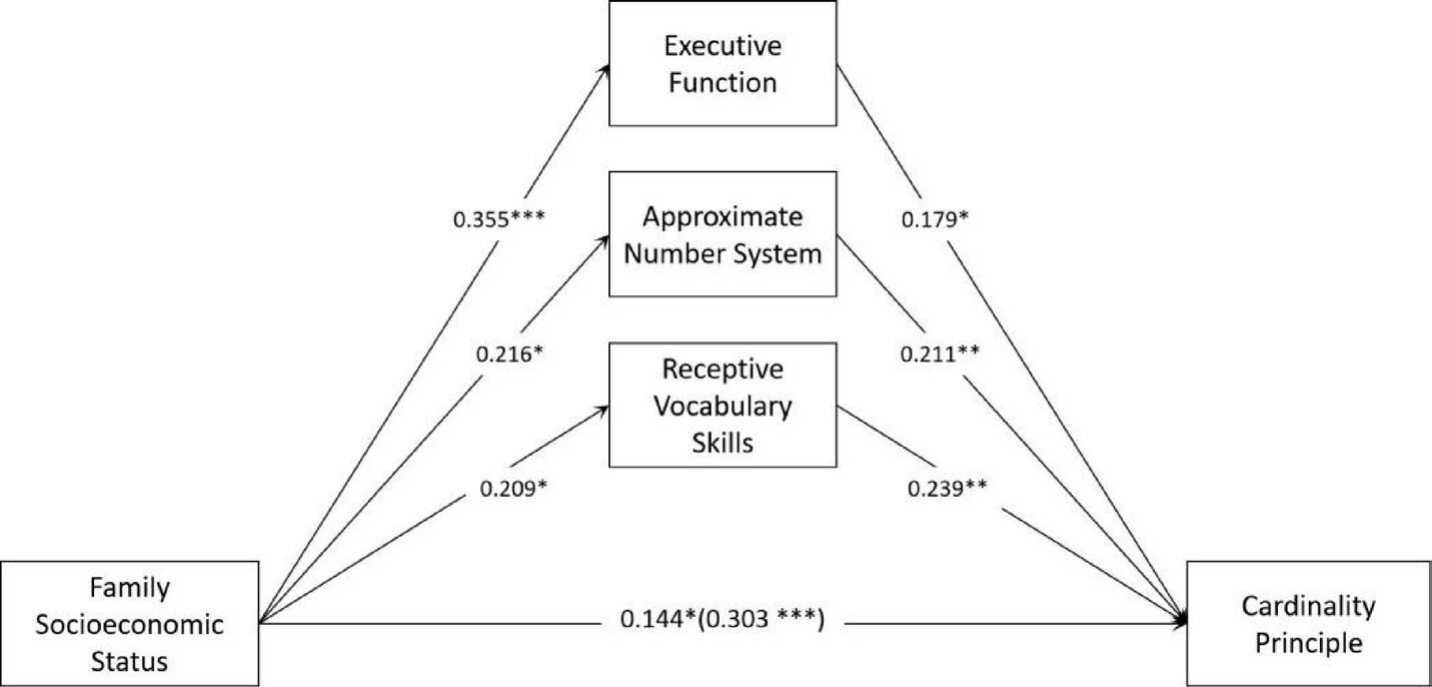
Model of the Mediating Role of Young Children’s EF, ANS, and RVS between SES and CP.

The indirect relationship was tested using a bootstrap method with 5,000 random samples. A significant mediation effect was indicated if the confidence interval did not include 0. The test results show (Table 5) that the direct path effect value for SES → CP is 0.144 (95%CI =[0.028, 0.013], not including 0). This SES can directly predict the CP. The indirect path effect value for Path 1 is 0.064 (95%CI =[0.015, 0.129], not including 0). This suggests that the EF is partially associated with the relationship between SES and the CP, with the indirect link representing 0.064/0.303 = 21.12% of the total association. The indirect path effect value for Path 2 is 0.046 (95% CI = [0.009, 0.095], excluding 0). This suggests that the ANS is partially associated with the relationship between SES and the CP, with the indirect link representing 0.046/0.303 = 15.18% of the total association.The indirect path effect value for Path 3 is 0.050 (95% CI = [0.009, 0.099], not including 0). This suggests that RVS is partially associated with the relationship between SES and the CP, with the indirect link representing 0.050/0.303 = 16.50% of the total association. Consequently, mediating roles 1, 2, and 3 were significant, with a total indirect association of 0.159 (95% CI =[0.077, 0.247], not including 0). Together, they explained 0.159/0.303 = 52.48% of the total association. A mediating model of EF, ANS, and RVS is shown in Fig. 2.

**Table 5:** Bootstrap analyses of the mediation effect.

	Effect	BootSE	Bias-corrected 95% CI		Effect Size ratio
			Lower limit	Upper limit	
Total effect	0.303	0.059	0.000	0.132	100%
Direct effect	0.144	0.053	0.028	0.013	47.52%
Total indirect effect	0.159	0.043	0.077	0.247	52.48%
Indirect effect 1(EF)	0.064	0.029	0.015	0.129	21.12%
Indirect effect 2(ANS)	0.046	0.022	0.009	0.095	15.18%
Indirect effect 3(RVS)	0.050	0.023	0.009	0.099	16.50%

*Notes.* ^*^p < 0.05, ^**^p < 0.01, ^***^p < 0.001.

## Discussion

The findings of this study contribute to the understanding of how SES, EF, ANS, and RVS serve as predictors of young children’s CP, particularly within the context of Chinese culture. This study is the first to explore the mediating role of EF, ANS, and RVS between SES and CP in young children. This study provides important theoretical support for exploring cognitive development in young children and also provides the scientific basis for practical guidance and intervention strategy design in related fields.

It should be noted that the present findings help to address several inconsistencies in the existing literature. While previous studies have reported different conclusions regarding the role of EF, ans, and RVS in CP development, our parallel mediation model provides a potential explanation for these differences. First of all, methodological differences are an important source of divergent results. Earlier single-mediator studies (e.g., examining only EF or ANS) may be biased by ignoring compensatory mechanisms in the cognitive domain. For example, children with weaker ANS may compensate for disadvantages by strengthening executive control strategies (e.g., finger counting). Second, cultural specificity explains the weight differences in mediation pathways. Chinese culture emphasizes the importance of education and collectivist values, which may influence how families invest in and support their children’s education. For example, Chinese families usually place a high value on their children’s academic achievement, which may be reflected in their enthusiasm to provide their children with abundant learning resources and to participate in educational activities. The Chinese education system focuses on the development of math and language skills in the early education stage, which may have promoted the development of young children in EF, ANS and RVS, thus influencing their understanding of the CP. Unlike Western studies that emphasize the mediation dominance of language, this study found that the mediating strength of EF in Chinese children is equivalent to language, which is consistent with the characteristics of East Asian education that focuses more on executive function training^98^. Such cultural differences suggest that socioeconomic predictions are context-dependent, and the possible mediators must be reexamined in a cross-cultural framework.

### Family socioeconomic status as a predictor of young children’s cardinality principle

The results indicate that SES significantly predicts young children’s performance on CP tasks, affirming the first research hypothesis. Higher SES is associated with better outcomes in CP tasks, aligning with ecological systems theory, which posits that interconnected environmental factors significantly influence cognitive development^100^.

According to the Family Investment Model, families with higher SES typically provide better educational resources, including high-quality early education programs, learning materials, and extracurricular activities^17,101,102^. Such resources enhance children’s mathematical learning opportunities, directly impacting their CP development. Moreover, parents from higher SES backgrounds often engage more actively in their children’s education, recognizing the importance of early cognitive stimulation. This engagement is supported by research indicating that a 10% increase in household income correlates with a significant rise in parental investment in early childhood education^103^. Positive parent-child interactions, such as number recognition and counting during everyday activities, provide enriching learning experiences and foundational mathematical skills^104^. Furthermore, children from high SES families generally benefit from better health care and nutrition, fostering physical health and cognitive development, which are crucial for CP^105,106^.

### The mediating role of executive function

The findings also highlight the partial mediating role of EF in the relationship between SES and CP, supporting the second research hypothesis and corroborating previous studies^10^. EF is critical for behavioural regulation, attention, and cognitive flexibility, essential for mathematical learning^27,28^. Children utilize inhibitory control to focus on relevant information and ignore distractions during math tasks. Additionally, motor-assisted counting, such as using fingers, aids in maintaining attention and reducing cognitive load^107^.

Families with high SES typically provide rich cognitive stimulation that enhances EF development, while low SES families often lack resources for educational investment, impeding EF growth^17,108^. Furthermore, low-income families frequently face challenging environments characterized by instability and stress, which can adversely affect brain development and EF^109–111^. Consequently, impairments in EF can hinder children’s ability to process quantitative information and successfully engage in mathematical tasks^112,113^.

### The mediating role of the approximate number system

This study demonstrated that the Approximate Number System (ANS) mediates the relationship between socioeconomic status (SES) and young children’s understanding of the cardinality principle (CP), thereby supporting Hypothesis 3. Consistent with previous research, our findings indicate a positive association between the accuracy of the ANS and young children’s proficiency in mastering CP^55,114–116^. The ANS, an innate numerical system, equips preschoolers with an intuitive grasp of quantity, enabling them to process object counts without complete symbolic knowledge. This foundational skill aids in comprehending the order and fundamental meanings of number words^53,117^.

The findings corroborate the Early Developmental Model of Mathematics proposed by Geary^54^which posits that young children initially rely on their innate ANS for quantity judgment. Subsequently, they map verbal number words and Arabic numerals onto this system to grasp the quantities these symbols represent. The greater the accuracy of their ANS, the more profound their understanding and mastery of the CP. Neuroimaging studies further underscore the centrality of the ANS in numerical cognition, revealing that regions such as the intraparietal sulcus are crucial for processing numerical information^118–121^.

Furthermore, research has shown that early exposure to mathematics, particularly through home-based activities, significantly enhances ANS acuity^122,123^. Children from middle- and upper-income families often receive early mathematical instruction, leading to superior ANS performance. In contrast, children from low-income families typically lack such exposure, limiting their ANS development due to resource constraints. Consequently, lower ANS accuracy can hinder their comprehension of CP.

### The mediating role of receptive vocabulary skills

The results of this study indicate that receptive vocabulary skills (RVS) partially mediate the relationship between SES and young children’s CP, validating Hypothesis 4. This aligns with previous findings highlighting language development deficits as obstacles to early math learning, especially for children from low-SES families^78^.

Neuroimaging research has identified that brain regions responsible for language processing, particularly within the language-dominant cortex, are also engaged when interpreting Arabic numerals^124,125^. As RVS plays a crucial role in language skills, its development significantly impacts children’s grasp of cardinal numbers, emphasizing the shared neural pathways between language and mathematics^126^. Vygotsky’s theory posits that while thinking and language have distinct origins, they eventually converge, with language structures shaping cognitive development^127^. In mathematics, this implies that robust language skills facilitate mathematical reasoning. According to Carey^128^children’s mastery of mathematical concepts requires proficiency in number names and their association with specific quantities, all dependent on strong RVS.

Families with higher SES tend to provide more material resources and engage in supportive interactions that enhance language development^17,109^. In contrast, lower SES families often experience constraints that limit verbal interactions and educational activities, adversely affecting RVS development^17,129,130^. Consequently, disparities in family SES manifest in young children’s understanding and mastery of CP, mediated by their RVS.

In addition, although age and gender were treated as covariates rather than focal variables, their roles deserve discussion. The findings suggest a significant positive correlation between age and CP. This conclusion is consistent with the results of previous studies^96^. With the growth as children age, their brains gradually mature, and the brain areas related to cardinality continue to improve so that their ability to understand numbers and quantities can be improved. According to Piaget’s theory of cognitive development, children are in the pre-operation stage, and their cognitive ability in this stage is mainly based on intuitive action thinking and concrete image thinking. With the growth of age, children’s thinking ability gradually transitions from simple perception and action operation to more complex logical thinking^131^. The zero sex effect of CP contrasts with previous reports of boys’ advantage in number tasks^97^. However, it is consistent with recent meta-analyses showing negligible sex differences in early cardinality understanding^132^. This phenomenon is because some scholars pointed out that this may be because children’s thinking levels, especially the concept of number thinking, have not yet differentiated. Thus, boys and girls have not yet shown significant differences in cardinality concept development.

### Contributions, limitations, and future directions

This study highlights the direct and positive influence of SES on young children’s CP and its indirect roles mediated by EF, ANS, and RVS. Compared to previous studies, our findings clarify the roles of SES, EF, ANS, and RVS in shaping CP, offering a fresh perspective on the cognitive development of mathematics in early childhood. Additionally, the study elucidates the mechanisms through which SES affects CP, providing empirical support for relevant theoretical models.

The results of this study have important implications for education policy and practice, especially in low SES communities. The study highlights the importance of cognitive skills in shaping early mathematical ability by highlighting the mediating role of EF, ANS, and RVS in the relationship between SES and CP. For children of low socioeconomic status, who often do not have access to resources to develop these cognitive skills, targeted interventions can be designed to bridge this gap. These may include EF training programs that enhance inhibitory control, working memory, and cognitive flexibility; Involves a comparable number of ANS activities; And enriched language environments to facilitate RVS, all of which can be integrated into early childhood education curricula. In addition, the study’s findings call for policy initiatives to address the root causes of SES disparities, such as providing financial support and educational resources for low-SES families, improving the quality of early childhood education in low-income areas, and promoting parent-child interactions that favour cognitive development. Finally, while most previous studies focused on the Western context, this study revealed the Chinese context and provided valuable data for cross-cultural comparisons.

However, this study has several limitations. Firstly, the cross-sectional design limits the ability to infer dynamic relationships among variables over time. Future research should employ longitudinal designs to explore these developmental trajectories. Secondly, the sample size was restricted to specific regions and social groups, potentially limiting the generalizability of our findings. Future studies should aim for a broader, multi-regional and multi-cultural representation to enhance the robustness of the conclusions. Third, the How Many and Give-N tasks used in this study mainly focus on children’s performance in specific counting and extraction situations, which may not fully cover children’s use of CP in daily life. In the future, a variety of different tasks can be considered to evaluate children’s CP more comprehensively. Fourth, as this study focused on multiple parallel mediation, the chain mediating role of EF, ANS, and RVS between SES and CP could be considered in the future. Finally, this study did not consider other factors that affect young children’s math cognition, such as access to educational resources, family environment, parenting style, and genetic factors. Future research should cover these variables to better understand the factors that influence mathematics cognition in young children.

## Conclusion

This study illuminates the mediating roles of EF, ANS, and RVS in the relationship between SES and young children’s understanding of the cardinality principle. It addresses a gap in the existing literature, particularly within the Chinese cultural context. By recruiting 130 children and their parents from various socioeconomic backgrounds and employing diverse measurement tools, we comprehensively assessed SES, EF, ANS, RVS, and CP mastery. The findings revealed that SES directly predicts young children’s CP and indirectly influences it through EF, ANS, and RVS. This research provides critical insights into how SES shapes young children’s understanding of CP by influencing their foundational cognitive skills, ultimately enhancing their mathematical comprehension.

## Data Availability

The datasets generated during and/or analyzed during the current study are available from the corresponding author on reasonable request.
